# Improved impedance to maladaptation and enhanced VCAM-1 upregulation with resistance-type training in the long-lived Snell dwarf (*Pit1^dw/dw^*) mouse

**DOI:** 10.18632/aging.203875

**Published:** 2022-02-03

**Authors:** Erik P. Rader, Marshall A. Naimo, James Ensey, Brent A. Baker

**Affiliations:** 1 Centers for Disease Control and Prevention, National Institute for Occupational Safety and Health, Morgantown, WV 26505, USA; 2 West Virginia School of Medicine, Division of Exercise Physiology, Morgantown, WV 26506, USA

**Keywords:** skeletal muscle, stretch-shortening contractions, plantarflexor muscles

## Abstract

Snell dwarf mice with the *Pit1^dw/dw^* mutation are deficient in growth hormone, prolactin, and thyroid stimulating hormone and exhibit >40% lifespan extension. This longevity is accompanied by compromised muscular performance. However, research regarding young (3-month-old) Snell dwarf mice demonstrate exceptional responsivity to resistance-type training especially in terms of a shifted fiber type distribution and increased protein levels of vascular cell adhesion molecule-1 (VCAM-1), a possible mediator of such remodeling. In the present study, we investigated whether this responsiveness persists at 12 months of age. Unlike 12-month-old control mice, age-matched Snell dwarf mice remained resistant to training-induced maladaptive decreases in performance and muscle mass. This was accompanied by retainment of the remodeling capacity in muscles of Snell dwarf mice to increase VCAM-1 protein levels and a shift in myosin heavy chain (MHC) isoform distribution with training. Even decreasing training frequency for control mice, an alteration which protected muscles from maladaptation at 12 months of age, did not result in the overt remodeling observed for Snell dwarf mice. The results demonstrate a distinct remodeling response to resistance-type exercise operative in the context of the *Pit1^dw/dw^* mutation of long-lived Snell dwarf mice.

## INTRODUCTION

The Snell dwarf mouse with a recessive mutation in *Pit1 (Pou1f1)*, an anterior pituitary transcriptional factor, results in combined anterior pituitary hormone deficiencies of growth hormone, thyrotropin, and prolactin has been an invaluable model to investigate lifespan extension [1]. The mouse model was first described almost a century ago and subsequently found to demonstrate compromised anterior pituitary development, muted development overall, and exceptional longevity >40% relative to littermates [2–4]. Delayed or slowed aging in regards to T cell function, collagen cross-linking, incidence of cataracts, resistance to cancer, and kidney disease were observed to be concomitant with the lifespan extension [2, 5, 6].

Previous research demonstrated that muscles of this longevity animal model are weak and demonstrate poor performance maintenance during activity at young age. Specifically, at 3 months of age for Snell dwarf mice, muscle quality (force normalized to muscle mass) was low and the capacity for isometric torque to be sustained in the minutes following contractions was especially compromised at values 20% of those for control littermates [7]. This outcome was not unexpected given the deficiency in growth hormone and, consequently, secondary deficiency in circulating insulin-like growth factor 1 (IGF-1)/insulin signaling [8–10]. Despite these deficiencies, however, muscles of young Snell dwarf mice responded to resistance-type training especially in terms of improvement in fatigue recovery capacity. Following the resistance-type exercise consisting of stretch-shortening contractions (SSCs), contractions consisting of a consecutive sequence of isometric, lengthening, and shortening contractions, 3 days per week for one month, muscles of Snell dwarf mice improved isometric torque following SSCs by 200% [7]. This was accompanied by muscle remodeling specific to Snell dwarf mice comprised of a shift in fiber type distribution to a slower phenotype. Furthermore, vascular cell adhesion molecule-1 (VCAM-1), a key mediator for the transmigration of monocyte and endothelial progenitor cells across the endothelium, was increased 4-fold [7, 11]. Such a response highlighted VCAM-1 as a candidate to consider as an intermediary for the fiber type remodeling which was observed. While this research provided insight in characterizing the responsiveness at young age in the presence of the *Pit1* mutation, whether this remodeling signature persists was not tested.

The purpose of the present study was to characterize SSC training-induced performance changes, VCAM-1 upregulation, and muscle fiber type remodeling for Snell dwarf mice in the context of both 3- and 12-months of age. The SSC protocol consisted of 80 maximally activated SSCs (8 sets of 10 repetitions) to the plantarflexor muscles of Snell dwarf mice and control littermates. Such a protocol was administered 3 days per week for one month and found to result in disparate outcomes depending on age and genotype. In supplemental testing regarding control mice, a decreased training frequency of 2 days per week was evaluated. Regardless of training exposure, muscles of control mice did not exhibit the overt alteration to myosin heavy chain (MHC) distribution and VCAM-1 protein levels observed for muscles of Snell dwarf mice. The results provide insight into the upregulation of VCAM-1 and shift in fiber type profile following resistance-type training as a potential compensatory mechanism in the context of pituitary hormone deficiency.

## RESULTS

### Frequent 3 day per week training initiated an age-dependent decline in muscle mass, maximal performance, and muscle quality for control mice while no such training-induced decrement was observed for 12-month-old Snell dwarf mice

**Figure 1:**
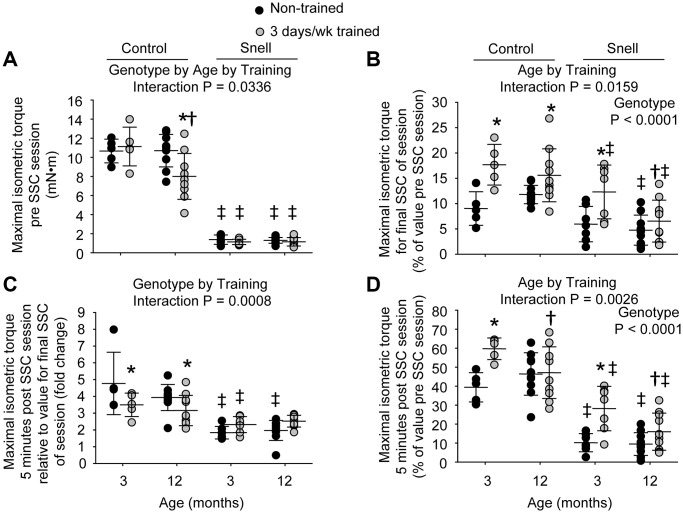
**Muscles of Snell dwarf mice were resistant to age-dependent maladaptation from frequent SSC training.** Training consisted of a frequency of 3 days per week for 1 month. (**A**) Maximal isometric torque output decreased with training for 12-month-old control mice while no such training-induced decrease was observed for Snell dwarf mice. (**B**) Torque depression by the final session SSC was generally reduced with training as assessed relative to pre SSC value. (**C**) Isometric torque recovery in the minutes following the SSC session was evaluated by comparing the 5 minute post SSC value with the final SSC value of the session. This recovery was unaltered with training for Snell dwarf mice unlike the reduced torque increase observed for control mice. (**D**) The overall isometric torque depression which persisted to 5 minutes post SSC session was reduced for 3-month-old mice with training regardless of genotype. Sample sizes were *N* = 5 to 10 per group. Dots represent raw values. Lines denote means ± SD. Relevant ANOVA interactions and main effects are noted. ^*^Different from non-trained value; ^†^Different from comparable 3-month-old value, ^‡^Different from comparable control value, *P* < 0.05.

At young age, Snell dwarf body weights were 30% of those for control mice (Table 1). Tibial lengths of Snell dwarf mice were 70% of control values (Table 1). In the non-trained condition, Snell dwarf muscles were small, weak, and exhibited compromised torque maintenance following SSC session. Snell dwarf absolute mass and normalized mass (normalized to tibial length) values for the plantarflexor muscle group were 21% and 30% of control values, respectively (Table 1). Quantitative analysis of muscle fibers in transverse revealed that individual gastrocnemius muscle fibers of Snell dwarf mice were also smaller in cross-sectional area, 37% of control value (Supplementary Table 1). Muscle performance was affected by genotype to an even greater extent than that of muscle size. Maximal isometric torque, peak dynamic torque, and work outputs for non-trained Snell dwarf mice were only 12% to 15% of control values (Figure 1A and Supplementary Figure 1A, 1B, and 1C). Performance was low even after normalizing maximal isometric torque to muscle size to assess muscle quality, 43% of control values (Table 1). Isometric performance was also evaluated for the isometric portion of the final SSC of the training session and 5 minutes post session to assess torque depression at the termination of SSCs, torque recovery during the minutes following SSCs, and the torque depression that persisted 5 minutes post session (Figure 1B, 1C, and 1D). Torque recovery in the minutes following the SSC session was compromised for muscles of Snell dwarf mice relative to control mice (Figure 1C). This low torque recovery for Snell dwarf mice was accompanied by a more pronounced extent of torque depression that persisted at 5 minutes post SSCs (Figure 1D).

**Table 1:** Body weight, tibial length, muscle mass, and muscle quality data for Snell dwarf and control mice following 3 days per week training.

	**Body weight (g)**	**Tibial length (mm)**	**Muscle mass (mg)**				**Normalized muscle mass (mg/mm)**				**Muscle quality (mN·m/mg/mm)**
			**Gastrocnemius**	**Plantaris**	**Soleus**	**Plantarflexor group**	**Gastrocnemius**	**Plantaris**	**Soleus**	**Plantarflexor group**	
**Control**											
3 months old											
Non-trained	29.0 ± 3.2	18.5 ± 0.3	130.9 ± 12.0	16.7 ± 3.3	7.3 ± 2.4	154.9 ± 16.0	7.06 ± 0.57	0.90 ± 0.17	0.39 ± 0.13	8.36 ± 0.79	1.28 ± 0.14
3 days/wk trained	29.5 ± 2.7	18.0 ± 0.8	128.0 ± 12.3	16.3 ± 3.5	8.4 ± 2.6	152.8 ± 11.3	7.10 ± 0.44	0.91 ± 0.20	0.47 ± 0.14	8.48 ± 0.35	1.31 ± 0.23
12 months old											
Non-trained	41.6 ± 4.2^†^	18.8 ± 0.4	123.9 ± 12.9	16.7 ± 1.9	8.5 ± 0.9	149.1 ± 14.1	6.57 ± 0.60^†^	0.88 ± 0.10	0.45 ± 0.05	7.91 ± 0.65	1.35 ± 0.15
3 days/wk trained	40.9 ± 6.0^†^	18.8 ± 0.6^†^	108.7 ± 12.1^*†^	15.1 ± 2.5	7.9 ± 1.2	131.7 ± 13.8^*†^	5.77 ± 0.55^*†^	0.80 ± 0.12	0.42 ± 0.06	6.99 ± 0.60^*†^	1.13 ± 0.27^*^
**Snell**											
3 months old											
Non-trained	9.0 ± 0.8^‡^	13.2 ± 0.5^‡^	27.6 ± 4.1^‡^	3.5 ± 1.1^‡^	1.7 ± 0.4^‡^	32.8 ± 5.1^‡^	2.09 ± 0.31^‡^	0.26 ± 0.09^‡^	0.13 ± 0.04^‡^	2.49 ± 0.39^‡^	0.55 ± 0.16^‡^
3 days/wk trained	8.9 ± 1.4^‡^	12.8 ± 0.6^‡^	23.8 ± 5.0^‡^	4.0 ± 2.4^‡^	1.4 ± 0.7^‡^	29.2 ± 6.3^‡^	1.85 ± 0.32^‡^	0.31 ± 0.18^‡^	0.11 ± 0.05^‡^	2.26 ± 0.42^‡^	0.52 ± 0.14^‡^
12 months old											
Non-trained	11.5 ± 2.0^‡^	13.0 ± 0.5^‡^	26.6 ± 3.0^‡^	4.0 ± 0.9^‡^	1.8 ± 0.6^‡^	32.4 ± 2.9^‡^	2.04 ± 0.23^‡^	0.31 ± 0.06^‡^	0.14 ± 0.05^‡^	2.49 ± 0.22^‡^	0.53 ± 0.11^‡^
3 days/wk trained	13.2 ± 3.1^†‡^	13.2 ± 0.4^‡^	25.3 ± 4.3^‡^	3.3 ± 0.8^‡^	2.0 ± 1.0^‡^	30.6 ± 4.3^‡^	1.92 ± 0.30^‡^	0.25 ± 0.06^‡^	0.15 ± 0.07^‡^	2.32 ± 0.31^‡^	0.50 ± 0.20^‡^

Values are expressed as means ± SD. Sample sizes were *N* = 5 to 10 per group. ^*^Different from comparable non-trained value. ^†^Different from comparable 3-month-old value. ^‡^Different from comparable control value, *P* < 0.05.

The 3 days per week training at young age resulted in a ubiquitous reduction in isometric torque depression that persisted 5 minutes post SSCs especially for Snell dwarf mice. Training improved this measure by 1.5-fold for control mice and 3.2-fold for Snell dwarf mice (Figure 1D). The reduction in torque depression for Snell dwarf mice was a result of diminished torque depression at the termination of the SSC session coupled with an unaltered torque recovery in the minutes following the session (Figure 1B and 1C). In contrast, the training-induced decrease in torque depression at SSC session termination for control mice was coupled with a lower torque recovery (Figure 1B and 1C). Other measures including muscle size, maximal isometric torque, peak dynamic torque, stretch work, shorten work, and muscle quality were unaltered by the frequent training for both young control and Snell dwarf mice (Figure 1A, Table 1, and Supplementary Figure 1A, 1B, and 1C). Despite the absence of overall muscle mass changes for Snell dwarf mice, training induced an increase of 1.3-fold in total muscle fiber number density (Supplementary Table 2). This was suggestive of a decrease in muscle fiber size since percentage of tissue composed of interstitium was unaltered with training (19.0 ± 1.9 vs. 15.7 ± 2.6 % non-cellular interstitium; 5.9 ± 0.8% vs. 5.4 ± 2.1% cellular interstitium, *P* > 0.05). When considering this along with the lack of change in absolute and normalized muscle mass, this suggested an increase in cross-sectional fiber number possibly due to hyperplasia or fiber splitting.

At 12 months of age, muscles of control mice maladapted to 3 days per week training while muscles of Snell dwarf mice remained resistant to such a response. For 12-month-old control mice, training induced a 12% reduction in absolute and normalized mass of the plantarflexor muscle group (Table 1). This age and genotype dependent response to training is indicated by an ANOVA interaction between genotype, age, and training (*P* = 0.0411) for normalized plantarflexor muscle mass. This reduction was due to mass loss primarily in the gastrocnemius muscle which also demonstrates an ANOVA interaction between genotype, age, and training (*P* = 0.0451) for normalized gastrocnemius muscle mass (Table 1). The muscle mass decreases coincided with a 18% smaller type IIb muscle fiber cross-sectional area with training (Supplementary Table 1). Isometric and dynamic performance measures were lowered by 20% to 25% with training (Figure 1A and Supplementary Figure 1A, 1B, and 1C). While isometric torque depression at SSC session termination decreased with training, torque recovery in the minutes post SSCs was reduced and thereby resulted in a lack of training effect for torque depression persisting 5 minutes post SSCs (Figure 1B, 1C, and 1D). Furthermore, muscle quality values were 16% depressed compared with non-trained values (Table 1). The decrements in muscle mass and performance were not accompanied by alterations in percentage of tissue composed of non-degenerative muscle fibers (86.7 ± 1.7% vs. 87.4 ± 1.8%, *P* = 0.847), degenerative muscle fibers (0.3 ± 0.1% vs. 0.0 ± 0.0%, *P* = 0.277), non-cellular interstitium (11.2 ± 1.3% vs. 11.2 ± 1.5%, *P* = 0.991), or cellular interstitium (1.8 ± 0.4% vs. 1.4 ± 0.4%, *P* = 0.736). An increase in percentage of tissue composed of central nucleated muscle fibers was observed, 24.9 ± 7.0% vs. 9.6 ± 4.9%, *P* = 0.0172. None of the training-induced alterations observed for the 12-month-old control mice including performance decline were present for Snell dwarf mice (Figure 1 and Supplementary Figure 1).

### Training-induced muscle remodeling in the form of alterations in fiber type distribution and VCAM-1 protein levels was especially prevalent for Snell dwarf mice across age groups

**Figure 2:**
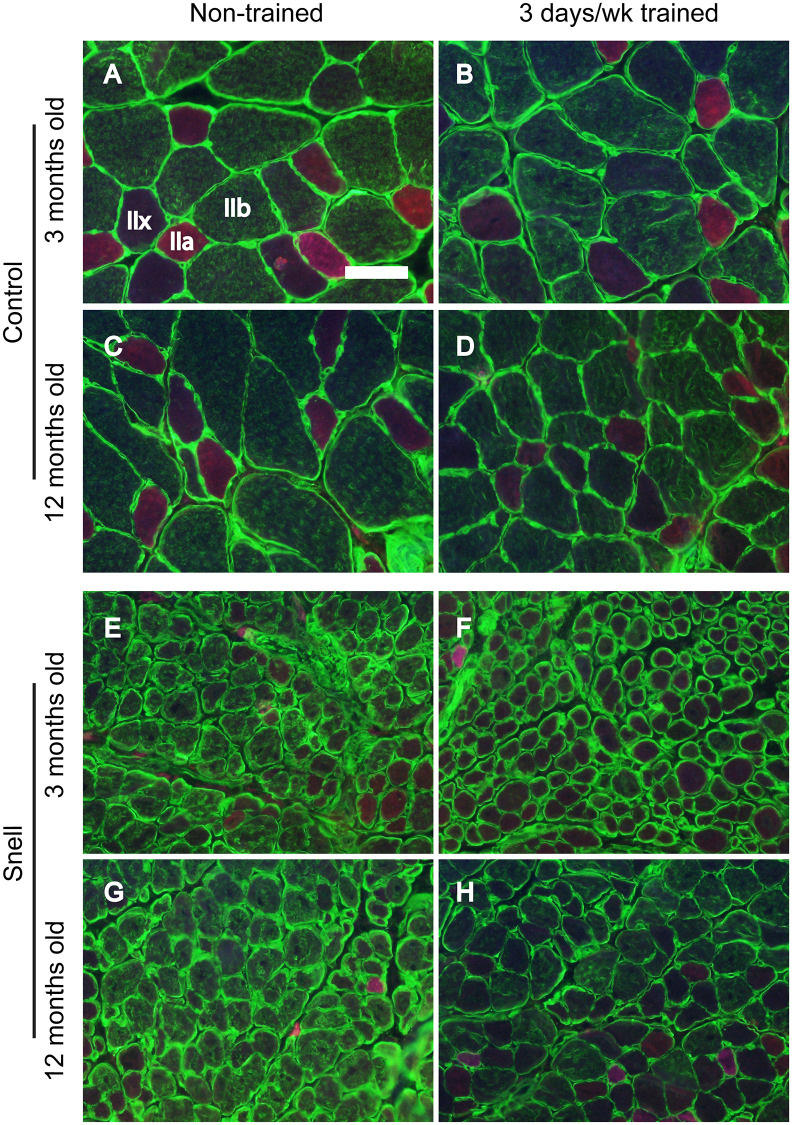
Fiber type immunofluorescence staining for muscles of control (**A**–**D**) and Snell dwarf (**E**–**H**) mice following 3 days per week training. Staining for laminin (green) and multiple MHC isoforms – IIb (green), IIa (red), and IIx (negative for staining) – are apparent in the images with shifting away from IIb to IIx with training unique to Snell dwarf mice. Scale bar = 50 μm.

**Figure 3:**
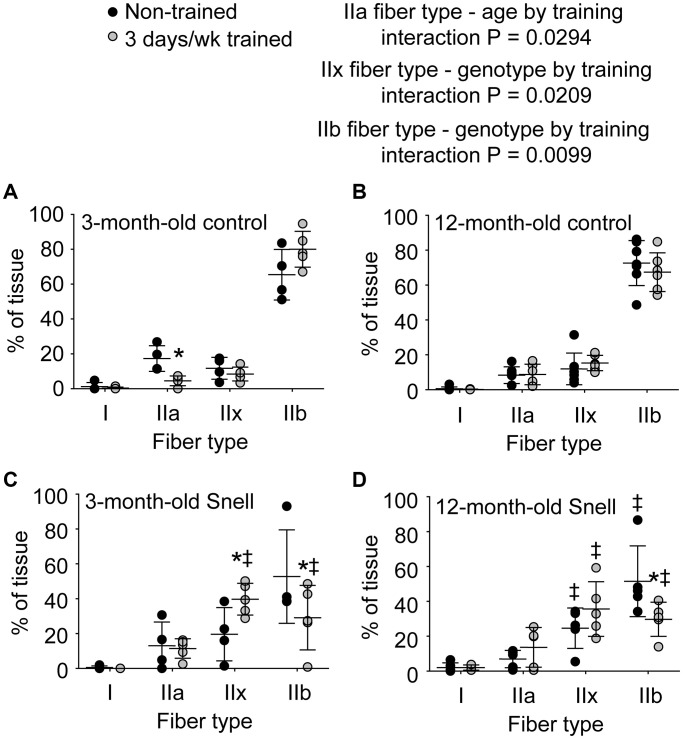
**Fiber type analysis demonstrated a 3 days per week training-induced redistribution of tissue to a slower phenotype exclusively for 3-month-old and 12-month-old Snell dwarf mice.** Percent of tissue composed of each fiber type for (**A**) 3-month-old control mice, (**B**) 12-month-old control mice, (**C**) 3-month-old Snell dwarf mice, (**D**) and 12-month-old Snell dwarf mice. Sample sizes were *N* = 4 to 7 per group. Dots represent raw values. Lines denote means ± SD. Relevant ANOVA interactions are noted. ^*^Different from comparable non-trained value, ^‡^Different from comparable control value, *P* < 0.05. No differences observed between age groups within genotype.

To further investigate the nature of tissue remodeling, fiber type and VCAM-1 distributions were evaluated. For Snell dwarf mice, the training response in torque depression reduction was accompanied by a shift in tissue composition away from type IIb muscle fibers to type IIx muscle fibers (Figures 2 and 3). The ability to shift fiber type distribution was preserved across age groups for Snell dwarf mice. Overall, these alterations in MHC isoform distribution were not accompanied by changes in cytochrome c oxidase (COX)/succinate dehydrogenase (SDH) histochemical double-labeling (Supplementary Figure 2 and Supplementary Table 3). Furthermore, for all the muscles analyzed, staining was dominated by light to dark brown COX label with no blue SDH staining evident. Such an outcome is consistent with normal COX activity (rather than defective COX synthesis or activity which allows for the blue reaction product of SDH to be apparent).

**Figure 4:**
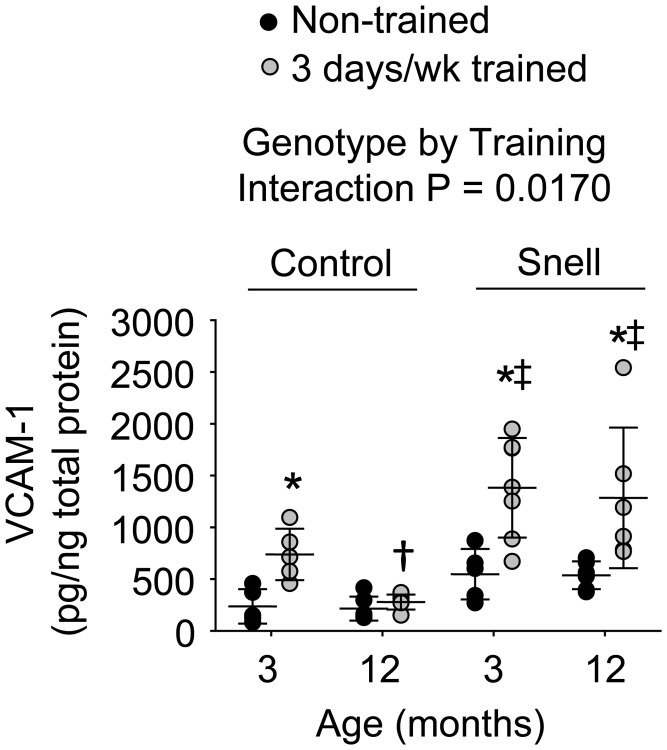
**VCAM-1 protein levels within muscle homogenates following 3 days per week training.** Exceptional VCAM-1 levels were reached in muscles of Snell dwarf mice upon training. Sample sizes were *N* = 5 to 7 per group. Dots represent raw values. Lines denote means ± SD. Relevant ANOVA interaction is noted. ^*^Different from comparable non-trained value, ^†^Different from comparable 3-month-old value, ^‡^Different from comparable control value, *P* < 0.05.

Immunofluorescence analysis was performed to determine the distribution of VCAM-1 in relation to capillaries as assessed by platelet endothelial cell adhesion molecule (CD31) localization (Supplementary Figure 3). ANOVA revealed that the main effects of age (*P* = 0.003) and genotype (*P* = 0.0008) impacted CD31^+^ node density (Supplementary Table 4). Relative to non-trained muscles of 3-month-old control mice, muscles of comparable Snell dwarf mice displayed 48% greater total CD31^+^ nodes per mm^2^ tissue (*P* = 0.0315). The factor of age was apparent between trained muscles of the different age groups of Snell dwarf mice with 32% higher CD31^+^ node density at 12 months of age versus 3 months of age (*P* = 0.0413). A more pronounced distribution of VCAM-1 within capillaries was indicated in Snell dwarf mice by the finding of a significant ANOVA main effect of genotype (*P* = 0.0273) regarding VCAM-1^+^/CD31^+^ nodes/mm^2^ (Supplementary Table 4). This was most apparent for muscles of trained 12-month-old Snell dwarf mice exhibiting a 3-fold greater value of VCAM-1^+^/CD31^+^ nodes/mm^2^ relative to values of age-matched trained control mice (*P* = 0.0117). No effect of training was observed for values of VCAM-1^+^/CD31^+^ nodes/mm^2^. Despite the lack of training effect on capillary localization of VCAM-1, protein levels of VCAM-1 were influenced by training (Figure 4). Training-induced VCAM-1 protein levels were the most extreme for Snell dwarf mice as indicated by a genotype by training interaction (*P* = 0.017) and the observation of training values 2-fold greater than those of control mice (Figure 4). Furthermore, training-induced increases in VCAM-1 protein levels persisted at 12 months of age exclusively for Snell dwarf mice (Figure 4).

### Less frequent 2 days per week training of control mice enhanced maximal performance for 3-month-old mice and induced no maladaptation for 12-month-old mice – all in the absence of VCAM-1 and fiber type distribution alterations

To explore whether decreasing training frequency for control mice would allow time for greater overt muscle remodeling to occur, 2 days per week training was tested. At 3 months of age, absolute muscle masses and normalized muscle masses were unaltered by the training (Supplementary Table 5). Maximal isometric torque, peak dynamic torque, stretch work, and shorten work increased by a range of 22% to 27% with training (Supplementary Figures 4A and 5). Similarly, training increased muscle quality by 19% in an age dependent manner for 3-month-old mice as indicated by an ANOVA interaction (*P* = 0.0221) between age and training (Supplementary Table 5). Unlike 3 day per week training, 2 day per week training did not induce any alterations in isometric torque depression or recovery for 3 month old mice (Supplementary Figure 4B, 4C, and 4D). While VCAM-1 protein levels were elevated to a moderate level (i.e. half of that for 3 days per week trained Snell dwarf mice) with training, no training-induced increase in VCAM-1^+^/CD31^+^ node density, COX/SDH staining, or alteration in fiber type distribution was observed (Supplementary Figures 6–10 and Supplementary Tables 6–9).

For 12-month-old control mice, 2 day per week training did not induce maladaptation. Absolute muscle masses, normalized muscle masses, and muscle quality values were unchanged with training (Supplementary Table 5). Maximal isometric and dynamic performance remained unaltered upon training (Supplementary Figures 4A and 5). Regarding torque depression and recovery following an SSC session, training lowered the extent of torque recovery in the minutes following SSCs but this was not coupled with alterations in torque depression at the termination of the SSC session or at 5 minutes post SSCs (Supplementary Figure 4B, 4C, and 4D). These performance outcomes were accompanied by an increase in percentage of central nucleated muscle fibers with training (18.3 ± 5.6% vs. 7.0 ± 2.7%, *P* = 0.047) and no training-induced alterations (*P* > 0.05) in percentage of tissue composed of non-degenerative muscle fibers (85.9 ± 1.4% vs. 87.3 ± 1.3%), degenerative muscle fibers (0.1 ± 0.1% vs. 0.0 ± 0.0%), non-cellular interstitium (12.1 ± 1.0% vs. 11.7 ± 1.2%), or cellular interstitium (1.9 ± 0.5% vs. 1.0 ± 0.3%). No differences with training were observed in fiber type distribution, COX/SDH staining, VCAM-1^+^/CD31^+^ node density, or VCAM-1 protein levels (Supplementary Figures 6–10 and Supplementary Tables 6–9). Overall, these findings demonstrated that decreasing training frequency improved performance outcomes of maximal performance while not inducing the overt muscle remodeling observed for 3 days per week trained Snell dwarf mice. This highlights the uniqueness of the training-induced alterations in MHC isoform and VCAM-1 protein levels in Snell dwarf mice.

## DISCUSSION

The present study demonstrates, in the context of *Pit1^dw/dw^* induced pituitary hormone deficiency, reduced maximal muscle performance coinciding with responsiveness to resistance-type SSC training and insusceptibility to training-induced maladaptation. This response in Snell dwarf mice was accompanied by remodeling in VCAM-1 protein levels and muscle fiber MHC isoform distribution thereby indicating these responses as possibly compensatory when anterior pituitary hormones are deficient. Overall, the results demonstrate for Snell dwarf mice that poor skeletal muscle performance can be addressed by SSC training and distinct remodeling without the typical extent of susceptibility to maladaptive overreaching/overtraining [12–18].

Based on associations observed between muscle quality and mortality as well as other age-dependent health outcomes, some have proposed that muscle quality is crucial for wellbeing throughout life [19–21]. Given this context, the finding of poor muscle quality and performance in general at baseline for the long-lived Snell dwarf mice even at young age in the present study could be considered unexpected. This finding may suggest that a lower threshold of muscle quality and performance is sufficient in laboratory conditions and/or that these muscle phenotypes are associative but not necessarily critical to longevity in some contexts. The study also furthers earlier work by demonstrating that diminished performance persists for Snell dwarf mice at 12 months of age thereby supporting the notion that this feature is long-term rather than transient [7]. Such an observation appears to be inherent in Snell dwarf mice due to disruption of growth hormone/insulin-like growth factor 1 (IGF-1)/insulin signaling [22]. This is not surprising since past studies reveal that both muscle strength and muscle quality (measures compromised for Snell dwarf mice in the present study) correlate with the growth hormone/IGF-1/insulin axis [8–10]. The present study is unique in demonstrating a long-term muscle performance deficit in the context of disrupted pituitary hormone/IGF-1/insulin signaling.

Despite poor baseline muscle performance, Snell dwarf mice demonstrated responsiveness to SSC training. Young Snell dwarf mice improved isometric torque and exhibited a 3.2-fold enhancement in the extent of isometric torque maintained 5 minutes post SSCs. This enhancement is meaningful even if poor muscle quality/performance can coexist with extended survival for Snell dwarf mice. While these muscle outcomes may be adequate in the laboratory setting such a phenotype maybe debilitating in a more natural setting. In this context, training-induced improvements in torque production and maintenance would be considered advantageous. This enhancement for Snell dwarf mice was accompanied by a shift from type IIb to IIx muscle fibers – an observation noted previously [7]. This shift in MHC isoform composition was not accompanied by an alteration in COX/SDH staining. A disconnect between MHC isoform and oxidative enzyme capacity is not unprecedented [23–26]. Furthermore, studies regarding muscles of mice demonstrate that improvement in fatigue management can occur with a shift in MHC isoform distribution despite an absence of a change in oxidative enzyme activity [23, 24]. These findings are consistent with the observation that myofibrillar ATP utilization varies with MHC isoform content with type IIb fibers being energy inefficient, type IIx and IIa fibers being moderately energy efficient, and type I fibers being the most energy efficient [27, 28]. Therefore, the improvement in performance during training for the Snell dwarf mice may have been influenced by the shift in MHC isoform composition irrespective of the unaltered COX/SDH profile. Concurrent with the shift in fiber type composition was an increase in VCAM-1 protein levels. VCAM-1 is a transmembrane adhesion protein of the immunoglobulin superfamily expressed on a wide range of cell types [29, 30]. In concert with selectins, VCAM-1 mediates the transmigration of cells such as monocytes and endothelium progenitor cells to transverse the endothelium of blood vessels into peripheral tissue [11]. Such a role of VCAM-1 for ushering cells into muscle tissue has the potential to remodel the extracellular matrix, myonuclei population, and, consequently, muscle fiber type since distinct extracellular matrix properties/myonuclei populations associate with specific fiber types [31, 32].

Even at 12 months of age in the present study, muscles of Snell dwarf mice retained remodeling capacity in terms of training-induced fiber type transformation and VCAM-1 protein upregulation. This was accompanied by the absence of maladaptation. This finding was not surprising given the slower fiber type profile at baseline for Snell dwarf mice (relative to control mice) and the further shift to even a slower fiber type distribution following training. Among different subtypes of type II fibers, slower fibers demonstrate less damage following eccentric contractions than faster fibers as illustrated by assessment of Z-band streaming in muscles of men following eccentric contractions [33]. This finding is also observed in muscles of rabbits [34]. Furthermore, investigation of individual muscle fibers isolated from men and women demonstrate that type II muscle fibers with different MHC isoform compositions also differ in susceptibility to contraction-induced force deficits (i.e. type II fibers with a slower MHC isoform composition exhibit lower force deficits relative to type II fibers with a faster MHC isoform distribution) [35]. Therefore, the initial high percentage of type IIx fibers in Snell dwarf muscles at baseline and the further shift of type IIb fibers to type IIx fibers upon training may decrease the propensity to accumulate myofibrillar damage and develop maladaptation.

An additional factor to consider when interpreting the differences observed between Snell dwarf mice and control mice in the present study is that Snell dwarf mice undergo delayed or impaired maturation (e.g., reproductive immaturity) [5, 36]. Therefore, the Snell dwarf-specific results could have been due to processes associated with impaired maturation. Future work in which Snell dwarf are treated with hormonal supplementation for robust induction of puberty has the potential to address whether maturation state was a key factor in in regards to the outcomes of the present study [5]. Despite the various differences we observed between the Snell dwarf mice and control mice, they both shared the result of a muted improvement in post SSC isometric torque values at 12 months of age following 3 days per week training. This illustrates that although Snell dwarf mice appear to exhibit a better-preserved phenotype across age groups in general (relative to the maladaptation observed for control mice), Snell dwarf mice do not escape the consequences of such age-dependent alterations entirely.

The maladaptive decrease in muscle mass and maximal isometric torque for 12-month-old control mice at adulthood following 3 days per week training in the present study demonstrated that an inappropriate resistance-type training protocol can become deleterious in a wild-type background early in life. This finding was consistent with a report regarding a similar protocol of 3 days per week SSC training for dorsiflexor muscles of rats [12]. While muscles of young 3-month old rats increased performance with training, muscles of adult 6-month old rats decreased positive work capacity [12]. Careful modulation of frequency of high-intensity training has been demonstrated to be effective at improving resistance training outcomes at advancing age in human subjects [37]. Likewise in research utilizing 30-month-old rats, decreasing SSC training frequency from 3 to 2 days per week resulted in muscle mass and performance enhancement rather than decrement [13, 14]. The present study confirms these findings thereby stressing the importance of recovery time by demonstrating no torque loss with training when 12-month-old control mice were exposed to 2 days per week training. Continued research regarding the modulation of training parameters for an expanded range of age groups is warranted to elucidate precise approaches to address distinct age-dependent muscular decrements.

## MATERIALS AND METHODS

### Animals

Male Snell dwarf (*Pit1^dw/dw^* ) mice and their age-matched normal-sized control littermates were F1 generation produced by bidirectional mating of DW/J *Pit1^dw/+^* (Jax# 000643) and B6.DW *Pit1^dw/+^* (Jax# 000772) mice. The investigators, equipment, and methods were the same as described in a previous report regarding SSC-trained plantarflexor muscles of young (3 months old at training onset) Snell dwarf mice and their control littermates [7]. Furthermore, there was no appreciable gap in testing between age groups (i.e., testing of 12-month-old cohorts began within a month of completing testing of 3-month-old cohorts). Consequently, performance data and tissue obtained from trained muscles of these 3-month-old mice [7] were utilized for comparison purposes with those of mice at 12 months of age (at training onset) in the present study. Groups of heterozygote (*Pit1^dw/+^*) and homozygote wild-type (*Pit1^+/+^*) mice were pooled and categorized as control mice [38]. All the mice tested in this study were provided NIH-31 Open diet (Teklad 7917). The mice were housed in an AAALAC-accredited animal quarters and at least one normal-sized female littermate was housed with Snell dwarf mice to provide warmth. Breeding and colony management of mice were done under specific-pathogen-free conditions with surveillance testing performed quarterly (test results were negative for pathogens). Mice were then transferred to a separate animal facility at least one week prior to SSC training and were housed in isolation in a dedicated rack of individually ventilated cages with HEPA filtration of supply air for the remainder of the study. All animal procedures were approved by the Centers for Disease Control and Prevention/National Institute for Occupational Safety and Health – Morgantown Institutional Animal Care and Use Committee in Morgantown, WV.

### SSC training

The SSC training protocol was based on a previous procedure demonstrated to induce muscle mass and performance gains in young rats [13, 14]. For each session, the mouse was anesthetized with isoflurane gas and placed in dorsal recumbency on a heated table. Plantarflexion muscle stimulation was performed via platinum electrodes placed subcutaneously to activate the tibial nerve and observed to induce plantarflexion. The left foot was then secured to a footplate of a dual mode muscle lever system (Whole Mouse Test System, 1300A, Aurora Scientific). Stimulation parameters were set for maximal contraction (8-V magnitude, 0.2-ms pulse duration, and 150-Hz frequency). Isometric and dynamic performances were evaluated prior to the 80 SSC training. Isometric performance consisted of a single maximal isometric contraction with the ankle at 90° (angle between tibia and foot). Dynamic performance was assessed by a single SSC test consisting of an isometric contraction for 200 ms at 110° ankle angle followed by rotation to 70° at 500° per second, returning to 110° at the same velocity, and holding at that angle for an additional 200 ms isometric contraction. For the single SSC test, peak torque and work values during the stretch and shorten phases of the SSC were determined. Work was determined by integrating the torque versus time curve and dividing by duration to calculate average torque and then multiplying by the angular displacement.

The training SSCs consisted of 8 sets (2-minute intervals between sets) with 10 SSCs per set (3-second intervals between SSCs) (Supplementary Figures 11 and 12). Muscles were maximally activated for each SSC at ankle angle 90° for 100 ms, rotated to 70° at 60°/s, returned to 90° at 60°/s, and deactivated 100 ms later. SSCs at a velocity of 60°/s induce a response characterized by the absence of overt muscle inflammation and degeneration in rodents days to weeks into training [7, 15, 39–41]. Isometric torque depression during and following the SSC session was assessed by comparing the torque for the isometric portion of the final SSC and the isometric torque 5 minutes following the SSCs, respectively, with pre SSCs maximal isometric torque. Torque recovery in the minutes following the SSC session was determined by dividing the isometric torque at 5 minutes post-SSCs by the value for the final SSC. Training consisted of one month of frequent 3 days per week training (Monday, Wednesday, and Friday) or less frequent 2 days per week training (Monday and Thursday) – two training schedules which resulted in distinct outcomes in previous research regarding old wild-type rats (i.e. 3 days per week training was maladaptive while 2 days per week training was adaptive) [13, 14]. Non-trained values for maximal performance measures and muscle tissue analyses were from mice exposed to the same procedures (e.g., time-matched anesthetization and assessment of maximal performance) and weekly schedule as trained mice with the exception that no training SSCs were administered. Initial and final performance values were determined by averaging the first and last week, respectively. Plantarflexor muscles were removed and weighed 72 hours after the final exposure. Tibia length was also measured and used to determine normalized muscle mass (muscle mass divided by tibia length). Muscle quality was calculated by dividing maximal isometric torque by normalized muscle mass of the plantarflexor muscle group. Each gastrocnemius muscle mid-belly portion was immersed in tissue freezing media and placed in cold isopentane (−160°C) for quantitative morphology/immunofluorescence while remaining tissue was stored for ELISA.

### Quantitative morphology

Gastrocnemius muscle tissue was cryosectioned at 12 μm thickness, stained with hematoxylin and eosin, and analyzed by a standardized stereological procedure [7, 14, 41, 42]. The investigator was blinded to sample identification. At 40X magnification, 5 fields (or maximum number of fields possible without overlap) were evaluated both right and left of the midline of the muscle section so that up to a total of 10 fields were assessed per muscle section. At each field, points of a 121-point 11-line overlay graticule (0.04 mm^2^ square with 100 divisions) were evaluated with each point identified as overlaying a muscle fiber (degenerative or non-degenerative) or interstitium (cellular or non-cellular). Degenerative muscle fibers were characterized as loss of contact with surrounding fibers, interdigitation of the sarcolemma by cellular infiltrates, and internalization of cellular infiltrates [42]. A point was categorized as overlying cellular interstitium when the point was over a nucleus in-between muscle fibers. A point overlaying a region lacking nuclei outside muscle fibers was characterized as cellular interstitium. Percent of muscle tissue comprised of non-degenerative muscle fibers, degenerative muscle fibers, centrally nucleated muscle fibers, cellular interstitium, or non-cellular interstitium were calculated as the percentage of points which overlaid each type of tissue relative to the total number of points. Number of muscle fibers per unit cross-sectional area was determined by counting the number of muscle fibers in which the topmost part of the fiber was within the graticule boundary. Mean muscle fiber area (μm^2^) was determined by dividing the percent of tissue comprised of muscle fibers by fiber number per unit area.

### Immunofluorescence

Gastrocnemius muscle transverse sections were stained for MHC isoforms using a previously described method [7, 41]. Sections were blocked (10% goat serum in PBS for 1 hour at room temperature) and incubated in a primary antibody cocktail overnight at 4°C - antibodies against MHC I (BA-F8; 1:10), MHC IIa (SC-71; 1:200), MHC IIb (BF-F3;1:200), and laminin (L9393;1:400). Sections were then exposed to secondary antibodies (Alexa Fluor from Life Technologies) for 2 hours at room temperature - 350 IgG2b goat anti mouse (A21140; 1:250), 594 IgG1 goat anti-mouse (A21125; 1:100), 488 IgM goat anti-mouse (A21042; 1:500), and 488 IgG goat anti-rabbit (A11008; 1:500). Analysis of Fiber type distribution was performed by a standardized stereological method with investigator blinded to sample identification [7, 41]. At both lateral and medial regions of the muscle section, non-overlapping images were captured and an overlay graticule (with 0.04 mm^2^ square boundary) was placed at the center of each image. Each point of intersection was categorized as overlaying MHC I (blue), MHC IIa (red), MHC IIb (green), MHC IIx (lacking staining), or interstitium. Percent of muscle tissue comprised of each fiber type was calculated as the percentage of points which overlaid each type of tissue relative to the total number of points. Number of muscle fibers of each type in which the topmost portion was within the boundary of the graticule were also counted. Total number of fibers counted divided by the total area sampled was calculated to determine the number of fibers per unit cross-sectional area. Mean muscle fiber area (μm^2^) was determined by dividing the percent of tissue comprised of a particular fiber type by the appropriate fiber number per unit area.

For analysis of VCAM-1 distribution, gastrocnemius muscle sections were fixed in HistoChoice (Sigma-Aldrich; H2904) for 45 minutes at room temperature, washed with phosphate-buffered saline (PBS, 3 × 5 minutes), and permeabilized in 0.2% Tween20 for 10 minutes. Sections were blocked for 1 hour (10% donkey serum in PBST at room temperature) and incubated overnight (4°C) with primary antibodies against VCAM-1 (PA5-47029 at 1:200 in blocking solution, ThermoFisher Scientific), laminin (L9393 at 1:400 in blocking solution, Sigma-Aldrich), and CD31 (550274, a marker of endothelial cells of blood vessels, at 1:50 in blocking solution, BD Pharmingen). This was followed by a 1 hour incubation with secondary antibodies (A11058 donkey anti-goat IgG Alexa Fluor 594, ThermoFisher Scientific, A21208 donkey anti-rabbit IgG Alexa Fluor 488, and A10039 donkey anti-rabbit IgG, ThermoFisher Scientific; each at 1:500 in PBST). The sections were mounted with a coverslip using Prolong Gold antifade mountant (P10144, ThermoFisher Scientific). With the investigator blinded to section identification, midpoint of the muscle section was identified and non-overlapping images were captured at the lateral and medial regions of the muscle section. An overlay graticule (with 0.04 mm^2^ square boundary) was placed at the center of each image and the investigator counted total number of CD31^+^ nodes (anatomical features encircled by laminin, adjacent to muscle fibers, and positive for CD31 staining thereby indicative of capillaries) and the subset of CD31^+^ nodes that were also positive for VCAM-1 staining (i.e. number of CD31^+^VCAM-1^+^ nodes) provided the topmost region of each node resided within the graticule boundary. Number of nodes were normalized by total muscle section area sampled.

### Histochemistry

Transverse cryostat sections (12 μm) of gastrocnemius muscle were exposed to a modified version of a standard COX/SDH double-labeling assay [43]. Sections were incubated with COX reaction mixture (1.4 mM diaminobenzidine tetrahydrochloride, 22 μM sucrose, 41 μM cytochrome *c,* and 20 μg/ml catalase in PBS, pH 7.6) for 1 hour at 37°C. The sections were rinsed with distilled water then then incubated with SDH reaction mixture (93 mM sodium succinate and 1.5 mM nitroblue tetrazolium in PBS, pH 7.6) for 1 hour at 37°C. With the investigator blinded to section identification, midpoint of the muscle section was identified. At 40X magnification, 5 fields (or maximum number of fields possible without overlap) were evaluated both right and left of the midline of the muscle section so that up to a total of 10 fields were assessed per muscle section. An overlay graticule (with 0.04 mm^2^ square boundary) was placed at the center of each field and the investigator visually classified each muscle fiber within the graticule boundary.

### ELISA

Gastrocnemius muscle tissue was homogenized in PBS (25 μl per mg of tissue) containing Halt Protease Inhibitor Cocktail (Thermo Scientific, 78438), centrifuged at 1500 rcf for 15 minutes at 4°C, and supernatant collected for ELISA analysis for VCAM-1 (Quanterix Custom Array Kit, #100-0286) per standard kit instructions. Aushon Cirascan Imaging System was used to obtain images of the arrays. Total protein was determined using a standard colorimetric bicinchoninic acid (BCA) protein assay (Pierce, Rockford, IL, USA).

### Statistical analysis

Data were analyzed using ANOVA (JMP version 11, SAS Institute, Inc., Cary, NC, USA) with the variable of animal identification as a random factor to account for repeated measures when appropriate. *Post hoc* comparisons were performed using Fisher’s least significant difference method. Data regarding type I fiber results were not normally distributed and, therefore, analyzed by Kruskal Wallis ANOVA on ranks. All data are expressed as means ± standard deviation (SD). *P* < 0.05 was considered statistically significant.

## Supplementary Materials

Supplementary Figures

Supplementary Tables
