# Supplementary material for: Improved impedance to maladaptation and enhanced VCAM-1 upregulation with resistance-type training in the long-lived Snell dwarf (Pit1dw/dw) mouse
Source: Aging (Albany NY). 2022 Feb 3;14(3):1157–85. doi: 10.18632/aging.203875 (PMC8876912; doi:10.18632/aging.203875)
Supplement: Supplementary Figures [file aging-14-203875-s001.pdf]

SUPPLEMENTARY FIGURES

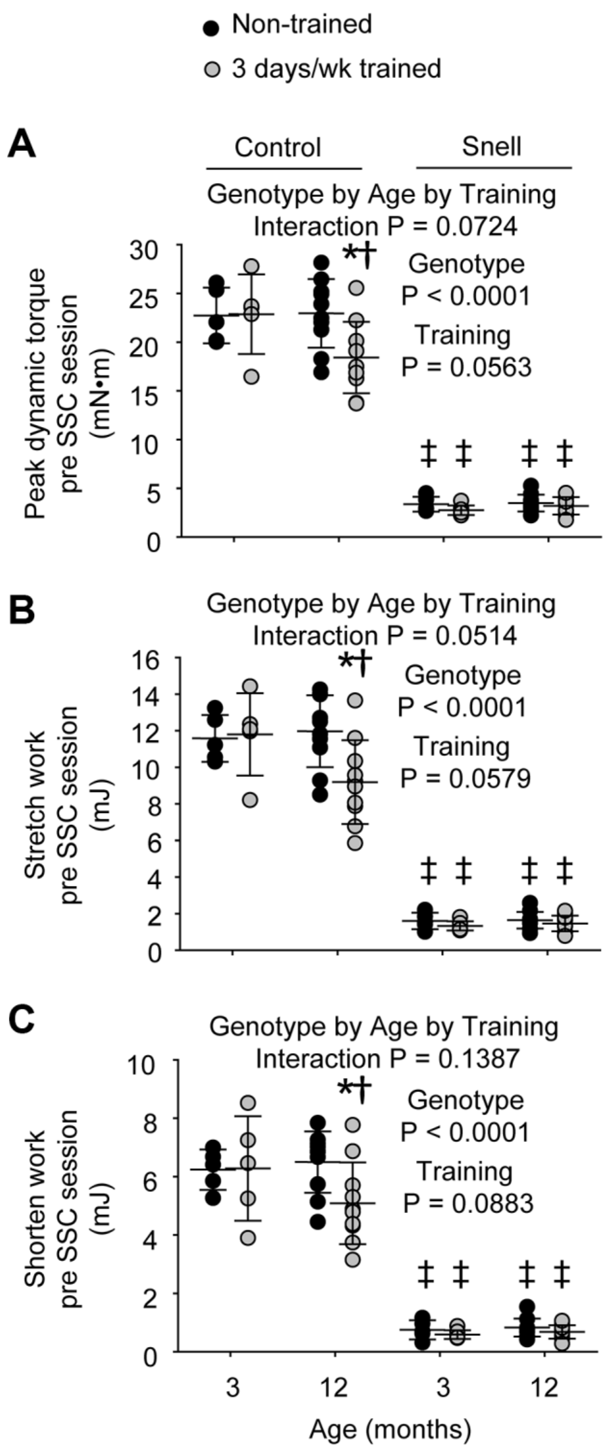

Supplementary Figure 1. Dynamic performance for non-trained and trained muscles of control and Snell dwarf mice. The dynamic measures of (**A**) peak dynamic torque, (**B**) stretch work, and (**C**) shorten work decreased with training for 12-month-old control mice while no such training-induced decrease was present for Snell dwarf mice. Sample sizes were *N* = 5 to 10 per group. Dots represent raw values. Lines denote means ± SD. Relevant ANOVA interactions and main effects are noted. *Different from non-trained value; †Different from comparable 3-month-old value, ‡Different from comparable control value, $p < 0.05$.

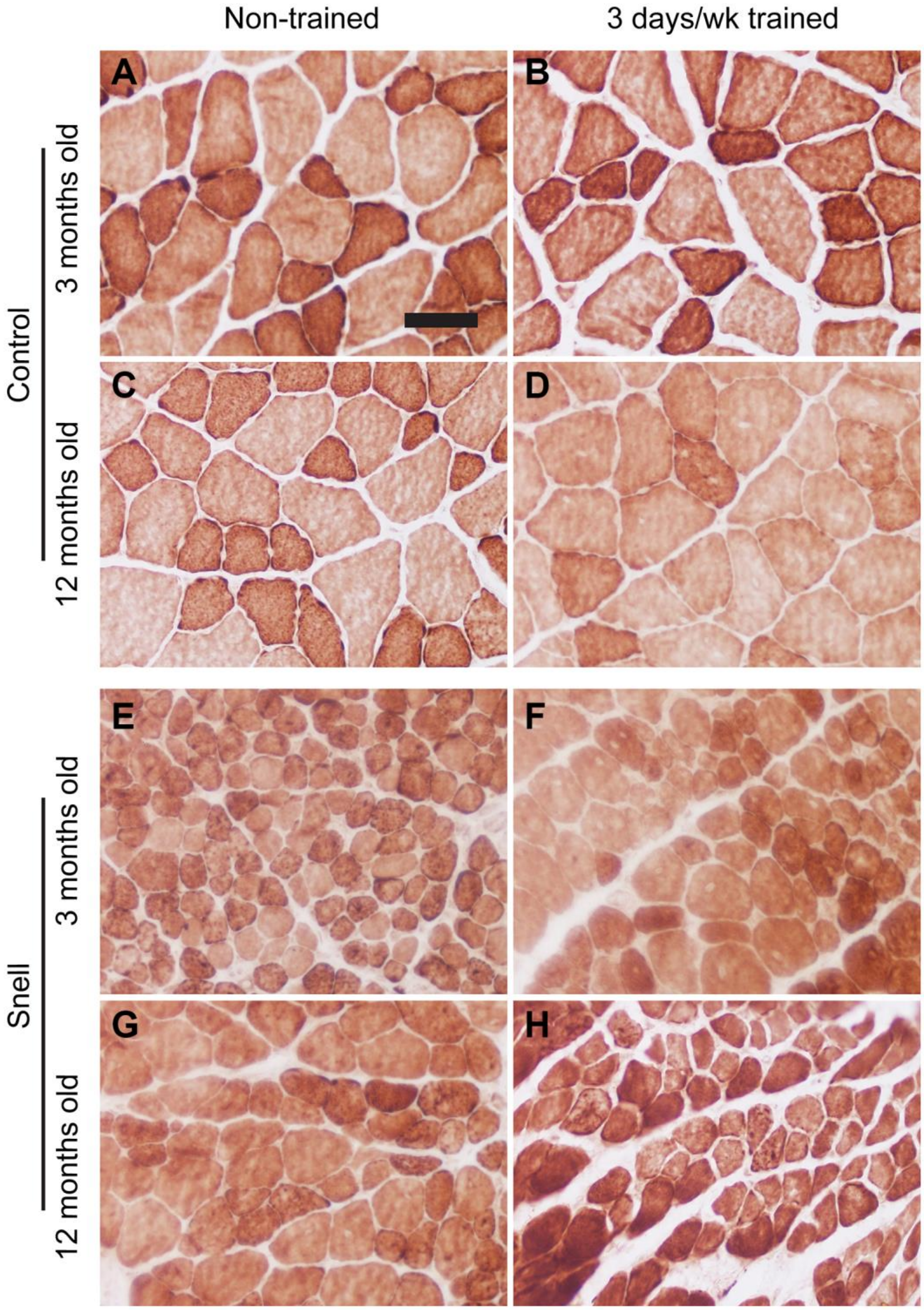

Supplementary Figure 2. COX/SOH labeling of muscles of mice following 3 days per week training. Images depict muscles of control (**A**–**D**) and Snell dwarf (**E**–**H**) mice. COX positive muscle fibers dominated displaying light and dark brown staining. No blue (COX deficient/SDH positive) fibers were observed. Scale bar = 50 µm.

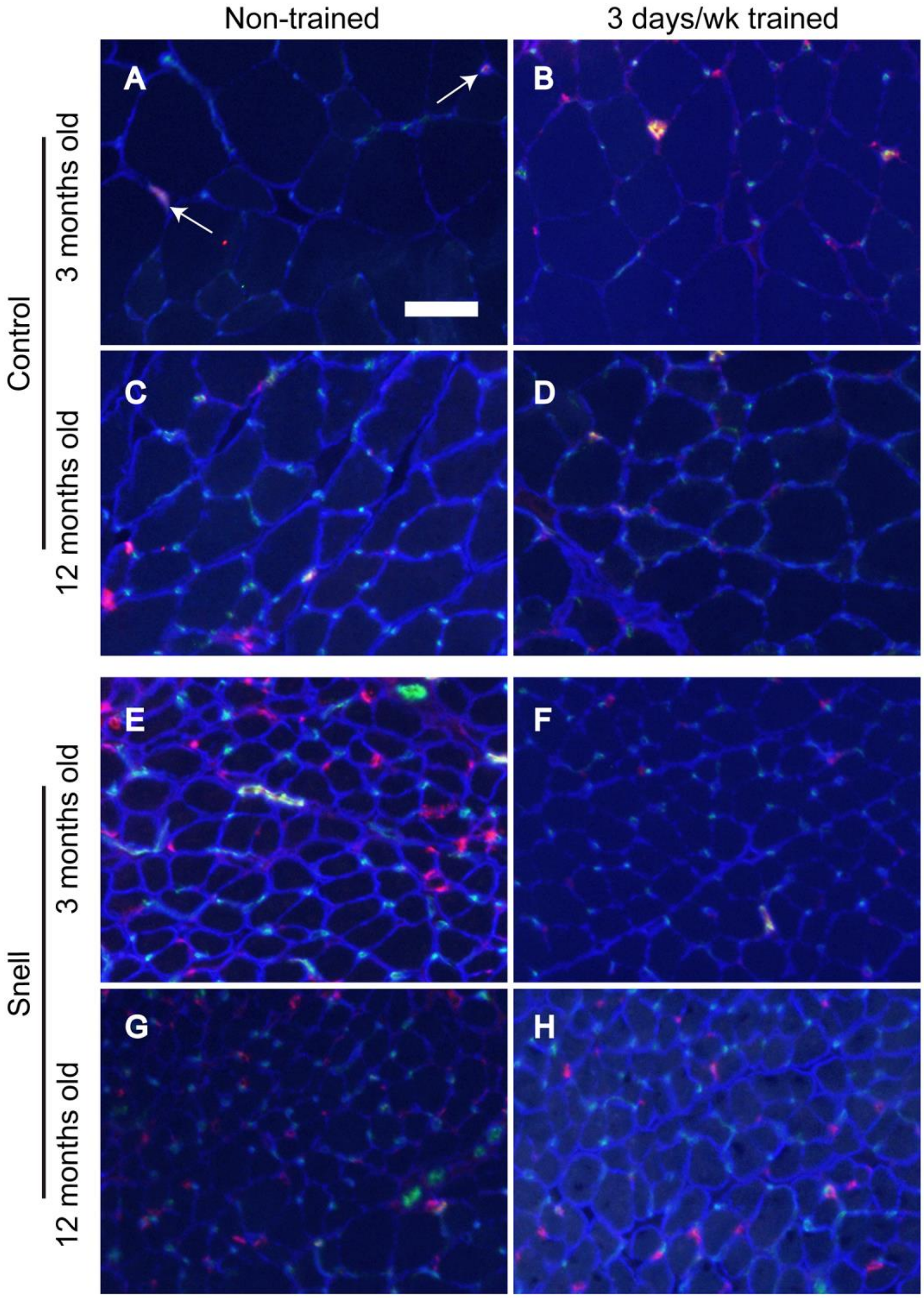

Supplementary Figure 3. Immunofluorescence staining for laminin (blue), CD31 (green), and VCAM-1 (red) in muscles of mice following 3 days per week training. Nodes (laminin encircled features adjacent to muscle fibers) were assessed for CD31^{+} and VCAM-1^{+}/CD31^{+} labeling (arrows depict VCAM-1^{+}/CD31^{+} examples in panel A) as an indicator of distribution of VCAM-1 within capillaries. Images were taken from muscles of control (**A**–**D**) and Snell dwarf (**E**–**H**) mice. Scale bar = 50 µm.

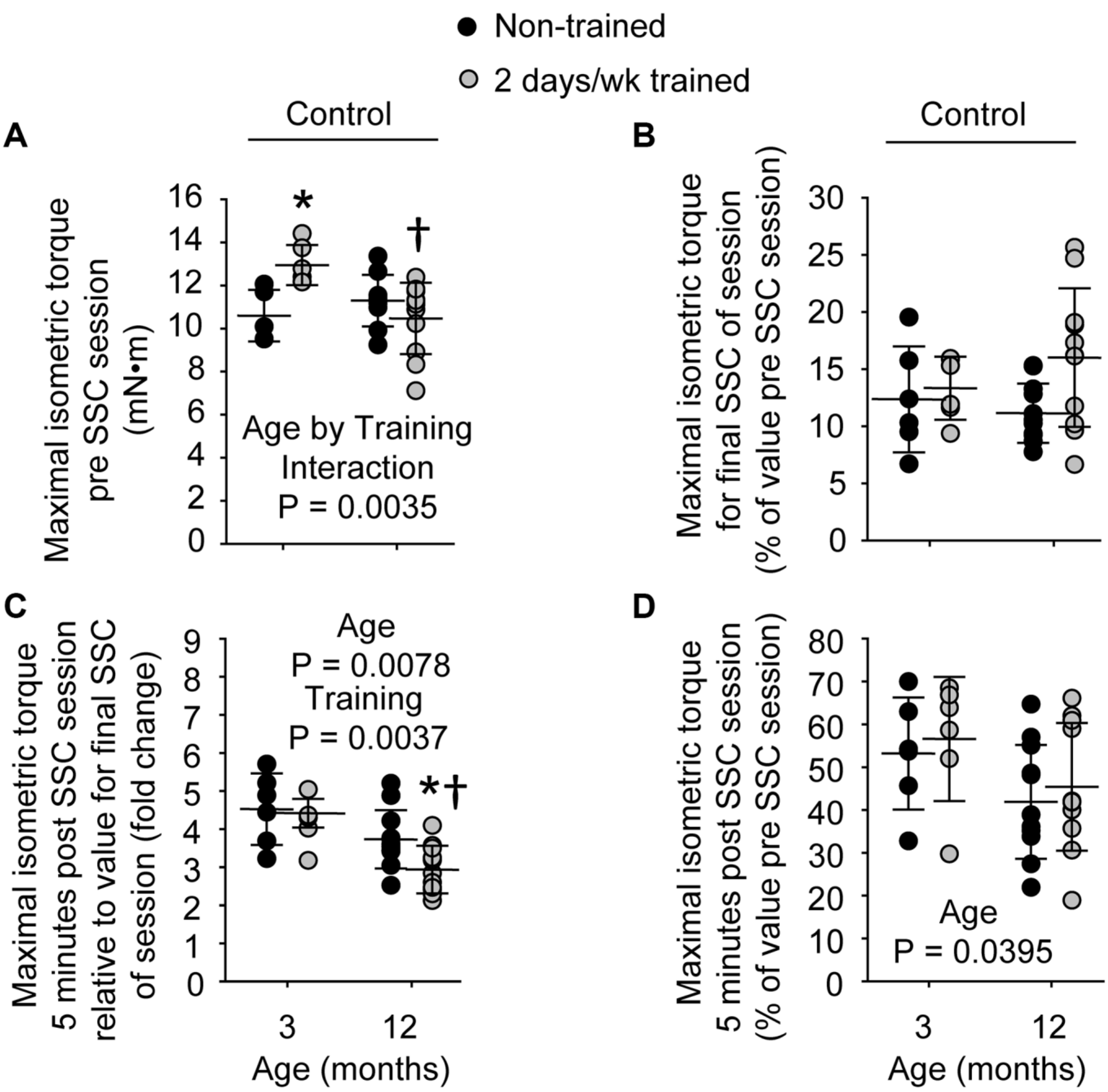

Supplementary Figure 4. Less frequent 2 days per week training enhanced adaptation for pre SSC session maximum isometric torque in 3-month-old control mice with no maladaptation in this measure for 12-month-old control mice. (**A**) Maximal isometric torque increased with training for 3-month-old mice and remained unaltered for 12-month-old mice. (**B**) Torque depression by the final session SSC was unaffected by training. (**C**) Torque recovery in the minutes following SSCs remained unchanged with training at 3 months of age and was reduced with training at 12 months of age. (**D**) However, overall isometric torque depression which persisted to 5 minutes post SSC session was unaltered by training for both age groups. (**B**) Sample sizes were N = 5 to 11 per group. Dots represent raw values. Lines denote means ± SD. Relevant ANOVA interactions and main effects are noted. *Different from non-trained value; †Different from comparable 3-month-old value, $p < 0.05$.

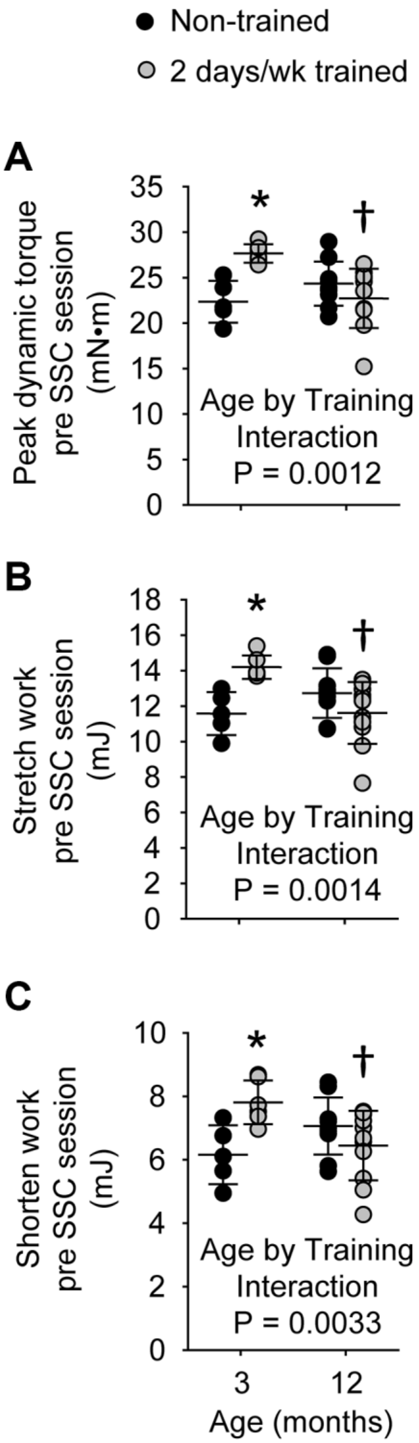

Supplementary Figure 5. Less frequent 2 days per week training enhanced adaptation for several dynamic performance measures in 3-month-old control mice with no maladaptation in 12-month-old control mice. The dynamic measures of (**A**) peak dynamic torque, (**B**) stretch work, and (**C**) shorten work were assessed. Sample sizes were N = 5 to 11 per group. Dots represent raw values. Lines denote means ± SD. Relevant ANOVA interactions and main effects are noted. *Different from non-trained value; †Different from comparable 3-month-old value, $P < 0.05$.

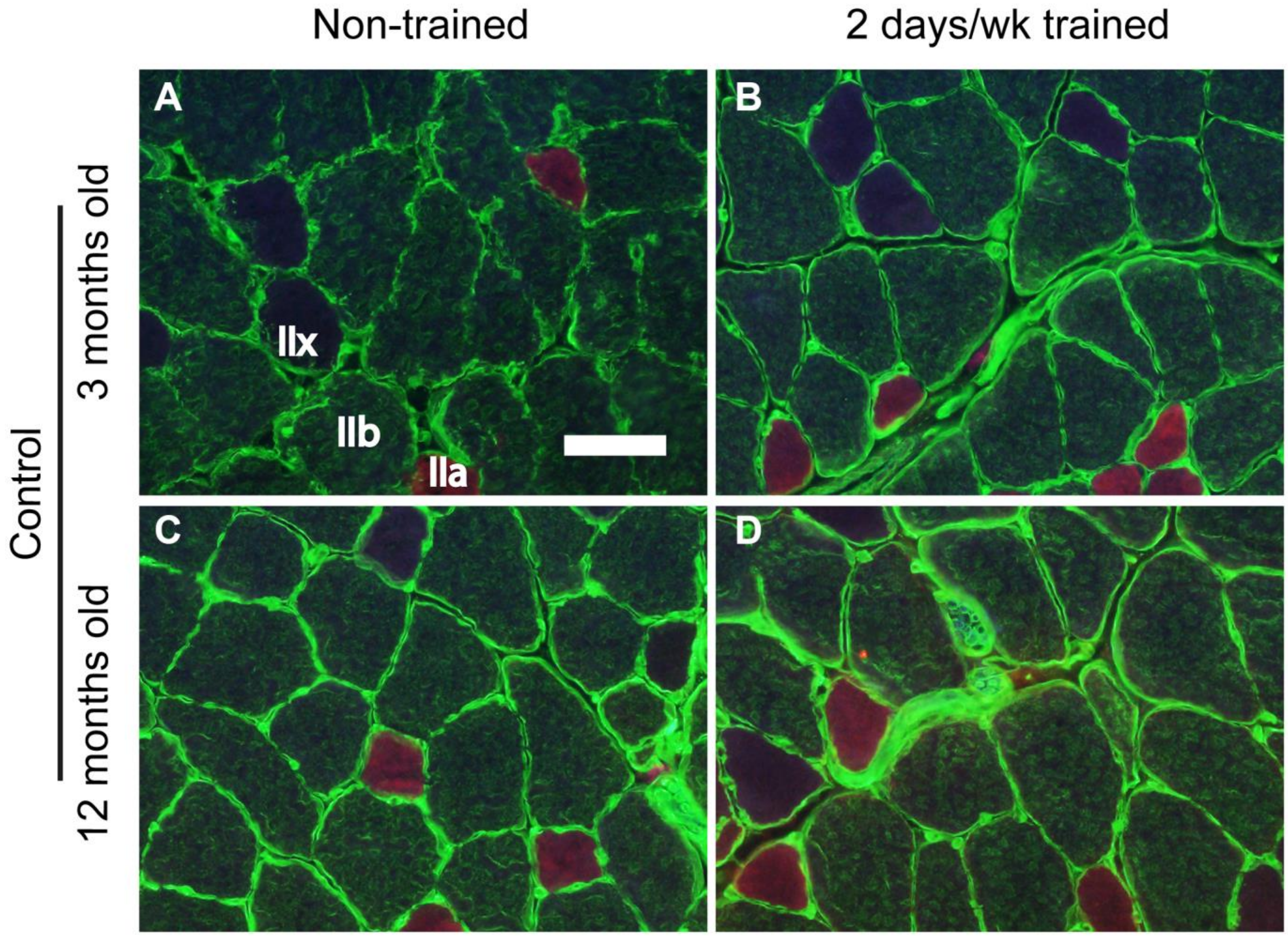

Supplementary Figure 6. Fiber type immunofluorescence staining for muscles of 3-month-old (**A**, **B**) and 12-month-old (**C**, **D**) control mice following non-training or 2 days per week training. Images depict immunofluorescence for laminin (green) and multiple MHC isoforms - IIb (green), IIa (red), and IIx (negative for staining). Scale bar = 50 µm.

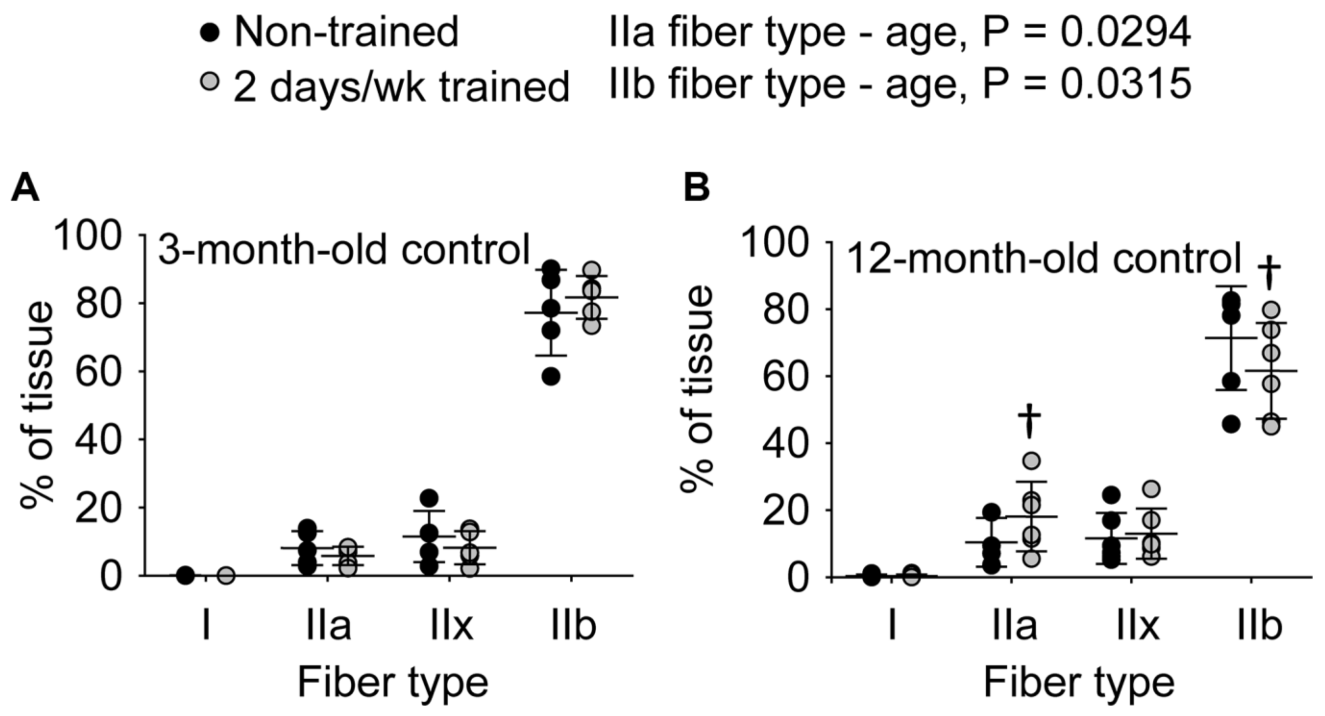

Supplementary Figure 7. Training 2 days per week for control mice had no effect on fiber type distribution relative to non-trained values. Percent of tissue composed of each fiber type for (**A**) 3-month-old control mice and (**B**) 12-month-old control mice. Sample sizes were *N* = 5 to 6 per group. Dots represent raw values. Lines denote means ± SD. Relevant ANOVA main effects are noted. †Different from comparable young value, $P < 0.05$.

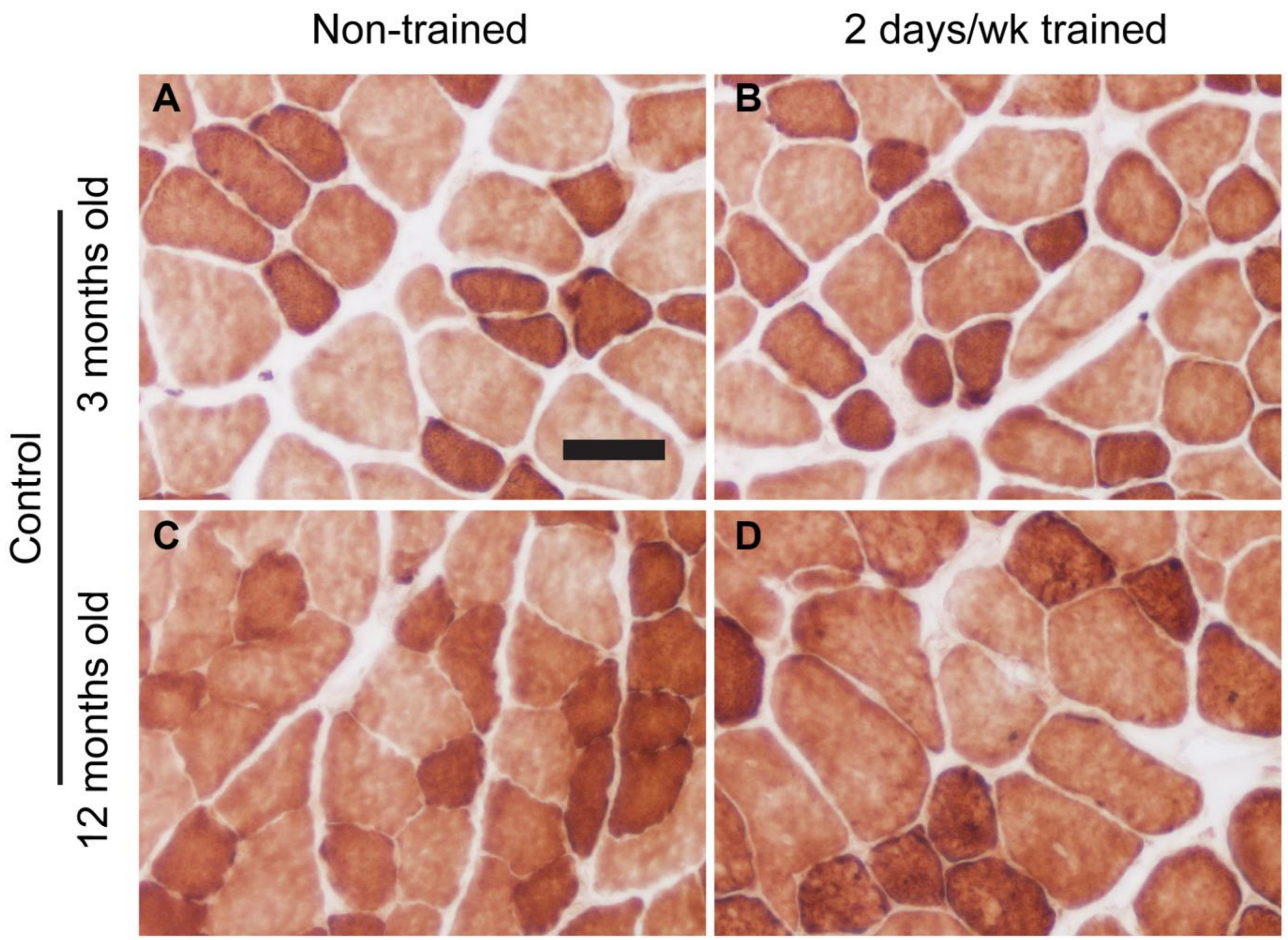

Supplementary Figure 8. COX/SOH labeling of muscles of control mice following 2 days per week training. Images depict muscles of 3 month old non-trained (**A**) and trained (**B**) muscles and 12 months old non-trained (**C**) and trained (**D**) muscles. COX positive muscle fibers dominated displaying light and dark brown staining. No blue (COX deficient/SDH positive) fibers were observed. Scale bar = 50 µm.

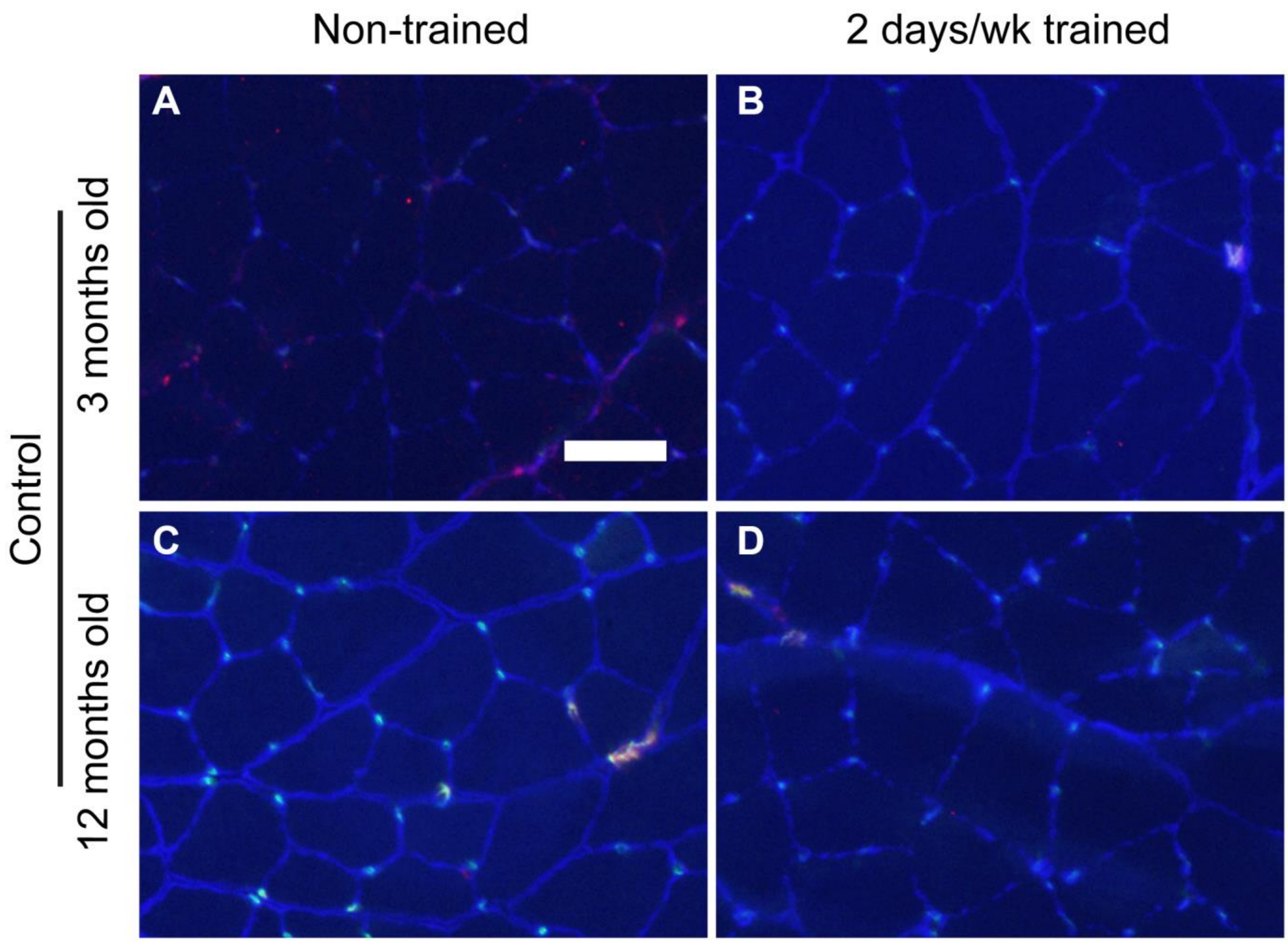

Supplementary Figure 9. Immunofluorescence staining for laminin (blue), CD31 (green), and VCAM-1 (red) in muscles of control mice following 2 days per week training. Images depict muscles of 3 month old non-trained (**A**) and trained (**B**) muscles and 12 months old non-trained (**C**) and trained (**D**) muscles. Scale bar = 50 µm.

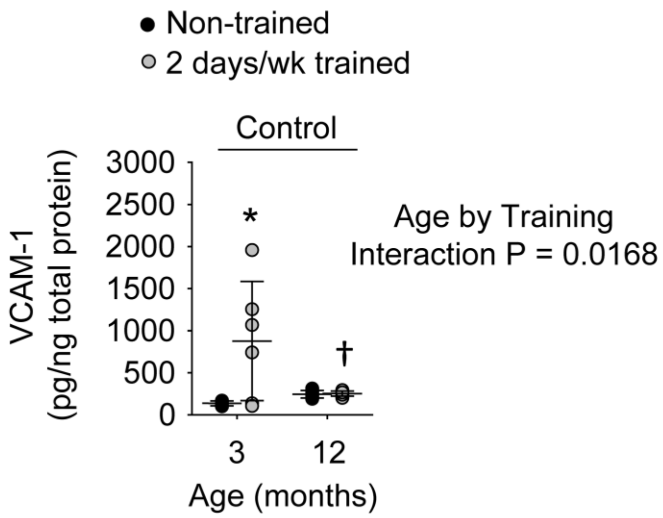

Supplementary Figure 10. VCAM-1 protein levels within muscle homogenates following 2 days per week training. Sample sizes were $N = 5$ to 6 per group. Dots represent raw values. Lines denote means ± SD. Relevant ANOVA interaction is noted. [*]Different from comparable non-trained value, [†]Different from comparable 3-month-old value, $P < 0.05$.

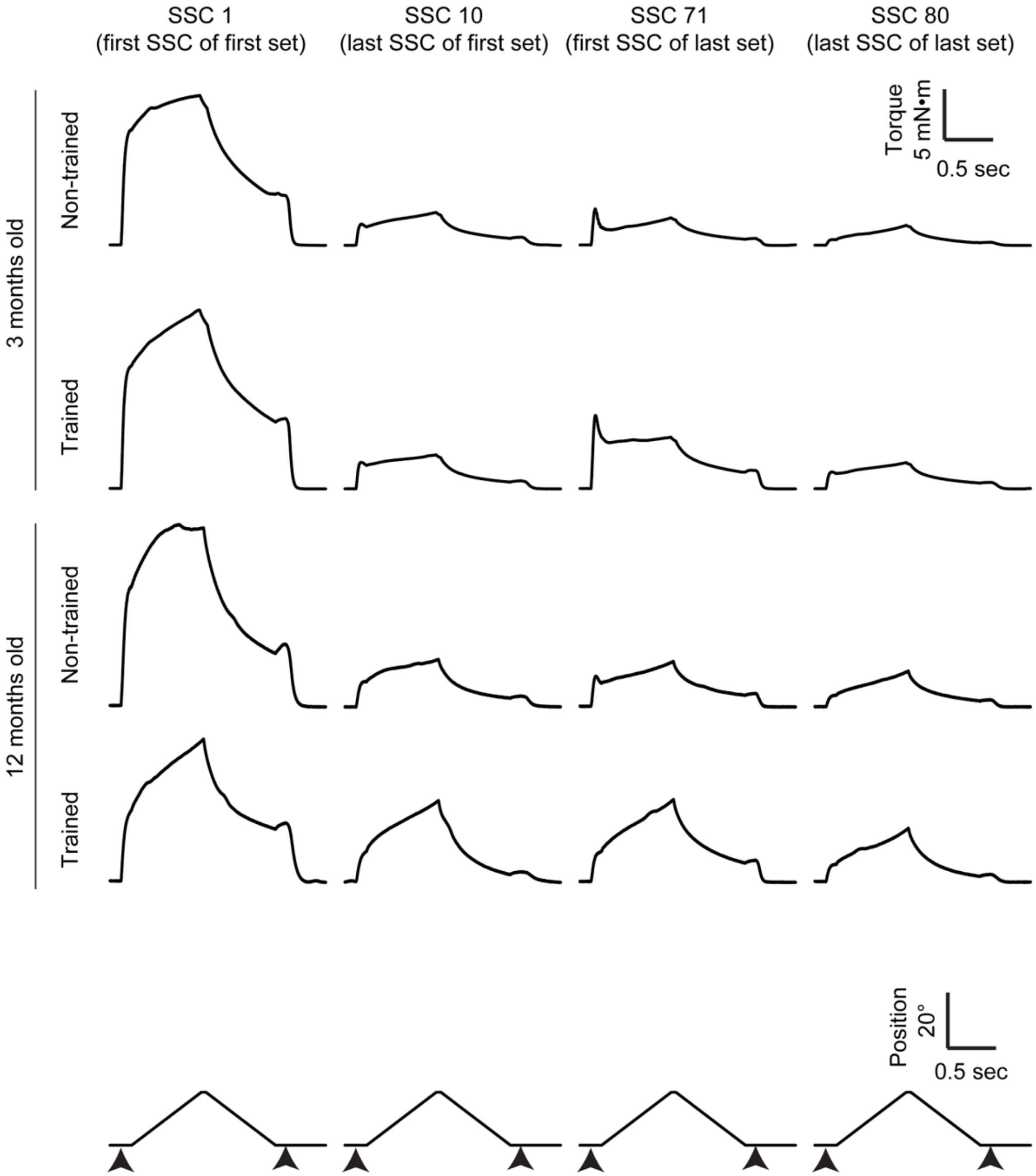

Supplementary Figure 11. Raw torque and position traces during a SSC session for non-trained and 3 day per week trained control mice. Traces for the first and last SSC of the first and last set are displayed. Each session consisted of a total of 8 sets with 10 SSCs per set and 2 minute rest intervals between sets. Arrows indicate when muscle activation began and ended while position traces display the 20° ankle rotation during each SSC. Each SSC consisted of a consecutive series of isometric, lengthening, shortening, and isometric contractions.

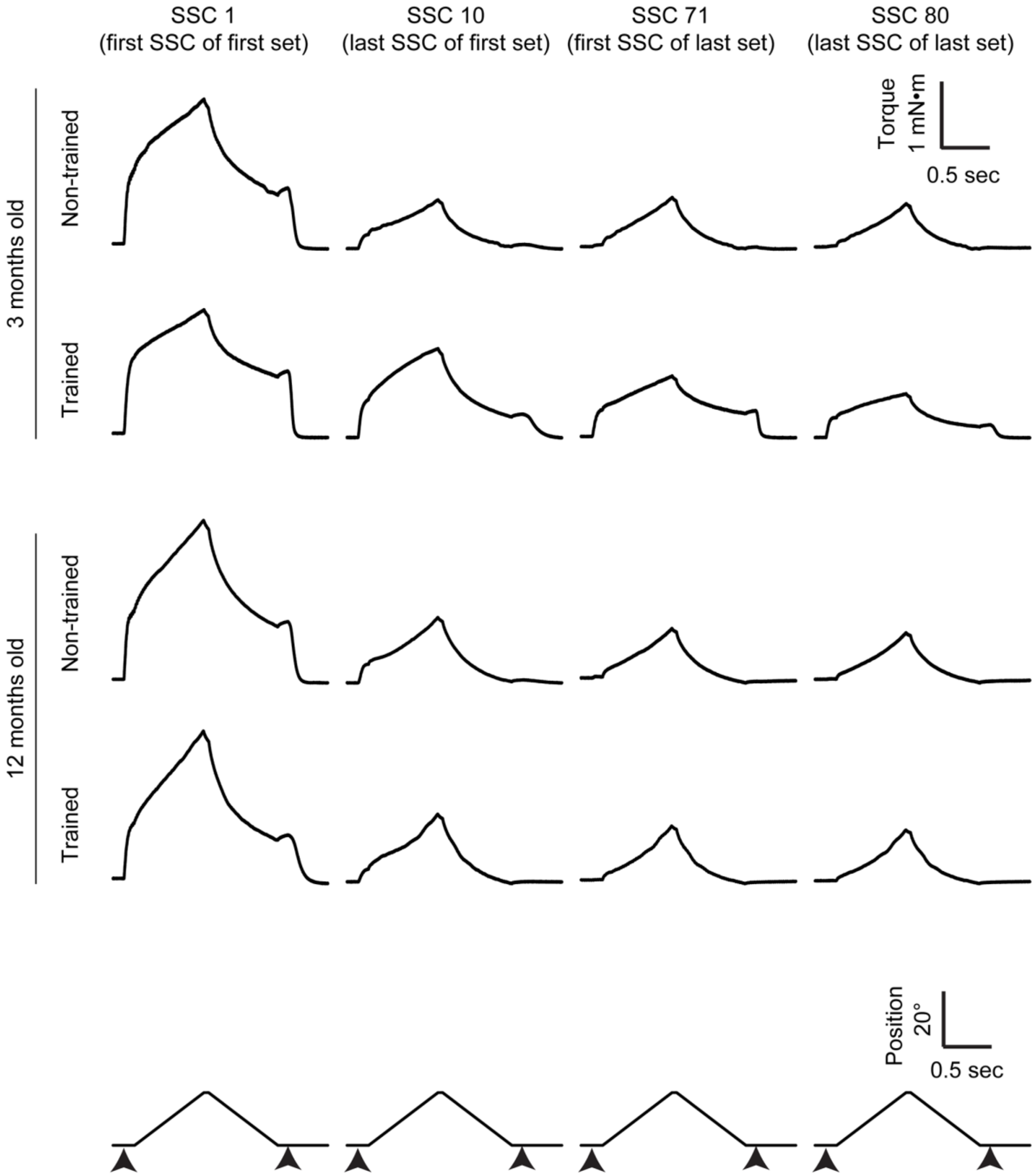

Supplementary Figure 12. Raw torque and position traces during a SSC session for non-trained and 3 day per week trained Snell dwarf mice. Traces for the first and last SSC of the first and last set are displayed. Each session consisted of a total of 8 sets with 10 SSCs per set and 2 minute rest intervals between sets. Arrows indicate when muscle activation began and ended while position traces display the 20° ankle rotation during each SSC. Each SSC consisted of a consecutive series of isometric, lengthening, shortening, and isometric contractions.
