# Supplementary material for: Improved impedance to maladaptation and enhanced VCAM-1 upregulation with resistance-type training in the long-lived Snell dwarf (Pit1dw/dw) mouse
Source: Aging (Albany NY). 2022 Feb 3;14(3):1157–85. doi: 10.18632/aging.203875 (PMC8876912; doi:10.18632/aging.203875)
Supplement: Supplementary Tables [file aging-14-203875-s002.pdf]

SUPPLEMENTARY TABLES

Supplementary Table 1. Muscle fiber cross-sectional area categorized by fiber type for control and Snell dwarf mice following 3 days per week training.

	Muscle fiber cross-sectional area μm²				
	I	IIa	IIx	IIb	Total
**Control**					
3 months old					
Non-trained	n/a	1060 ± 138	1773 ± 880	2504 ± 274	1939 ± 91
3 days/wk trained	n/a	822 ± 147	1636 ± 622	2471 ± 522	2193 ± 630
12 months old					
Non-trained	n/a	1114 ± 375	1540 ± 711	2676 ± 525	2179 ± 469
3 days/wk trained	n/a	1005 ± 650	1433 ± 414	2189 ± 233*	1756 ± 290*
**Snell**					
3 months old					
Non-trained	n/a	449 ± 9‡	418 ± 110‡	908 ± 339‡	718 ± 421‡
3 days/wk trained	n/a	497 ± 146	423 ± 150‡	525 ± 320‡	460 ± 135‡
12 months old					
Non- trained	436 ± 178	377 ± 123‡	440 ± 59‡	896 ± 178‡	657 ± 231‡
3 days/wk trained	540 ± 81	397 ± 184‡	560 ± 154‡	778 ± 187‡	583 ± 118‡

Values are expressed as means ± SD. n/a, data not available because of insufficient number of type I muscle fibers observed in samples to determine values. Sample sizes were N = 3 to 7 per group. *Different from comparable non-trained value. ‡Different from comparable control value, $P < 0.05$. No differences observed between age groups within genotype.

Supplementary Table 2. Number of muscle fibers per mm² categorized by fiber type for control and Snell dwarf mice following 3 days per week training.

	Number of muscle fibers per mm²				
	I	IIa	IIx	IIb	Total
**Control**					
3 months old					
Non-trained	0.0 ± 0.0	161.3 ± 57.8	64.6 ± 24.9	267.5 ± 84.3	493.3 ± 25.3
3 days/wk trained	1.7 ± 3.7	55.6 ± 32.8	59.8 ± 33.7	329.3 ± 43.4	446.3 ± 93.3
12 months old					
Non-trained	3.6 ± 9.4	7 1.1 ± 29.3	85.5 ± 76.7	282.0 ± 81.4	442.2 ± 79.2
3 days/wk trained	0.7 ± 1.7	103.6 ± 75.6	116.9 ± 57.5	309.6 ± 57.0	530.7 ± 75.9
**Snell**					
3 months old					
Non-trained	14. 1 ± 28.1‡	289.5 ± 307.7	523.0 ± 390.2‡	575.3 ± 131.2‡	1401.9 ± 468.8‡
3 days/wk trained	1.3 ± 2.8	252.3 ± 153.9	1078.9 ± 626.9*‡	520.4 ± 320.1‡	1852.9 ± 481.2*‡
12 months old					
Non-trained	39.3 ± 45.9†‡	182.8 ± 150.0	589.3 ± 30 1.6‡	572.3 ± 1 57.3‡	1383.5 ± 335.5‡
3 days/wk trained	36.0 ± 33.1†‡	275.3 ± 206.4	721.3 ± 442.7‡	391.6 ± 162.9	1424.1 ± 247.5†‡

Values are expressed as means ± SD. Sample sizes were N = 4 to 7 per group. *Different from comparable non-trained value. †Different from comparable 3-month -old value. ‡Different from comparable control value, $P < 0.05$.

Supplementary Table 3. Percentage of muscle fibers with dark brown staining (COX^{++} fibers) for control and Snell dwarf mice following 3 days per week training.

	%Cox^{++} fibers
**Control**	
3 months old	
Non-trained	41.2 ± 8.1
3 days/wk trained	45.2 ± 13.7
12 months old	
Non-trained	42.3 ± 11.5
3 days/wk trained	37.2 ± 14.1
**Snell**	
3 month s old	
Non-trained	45.7 ± 15.0
3 days/wk trained	39.1 ± 9.8
12 months old	
Non-trained	47.3 ± 8.6
3 days/wk trained	41.0 ± 9.9

Values are expressed as means ± SD. Sample sizes were N = 5 to 7 per group. No significant differences were observed.

Supplementary Table 4. Measures of total $CD31^{+}$ nodes and VCAM-$1^{+}CD31^{+}$ nodes for control and Snell dwarf mice following 3 days per week training.

	Total $CD31^{+}$ nodes/mm^2	VCAM-$1^{+}CD31^{+}$ nodes/mm^2
**Control**		
3 months old		
Non-trained	587 ± 86	96 ± 96
3 days/wk trained	656 ± 268	85 ± 47
12 months old		
Non-trained	759 ± 107	92 ± 73
3 days/wk trained	840 ± 214	112 ± 55
**Snell**		
3 months old		
Non-trained	866 ± 365‡	126 ± 70
3 days/wk trained	852 ± 129‡	114 ± 36
12 months old		
Non-trained	1040 ± 244‡	197 ± 107
3 days/wk trained	1128 ± 238‡	305 ± 214†‡

Values are expressed as means ± SO. Sample sizes were N = 4 to 7 per group. †Different from comparable 3-month-old value. ‡Different from comparable control value, $P < 0.05$.

Supplementary Table 5. Body weight, tibial length, muscle mass, and muscle quality data for control mice following 2 days per week training.

	Body weight (g)	Tibial length (mm)	Muscle mass (mg)				Nonmalizcd muscle mass (mg/mm)				Muscle qualiry (mN·m/ mg/mm)
			Gastrocnemius	Plantaris	Soleus	Plamarflexor group	Gastrocnemius	Plantaris	Soleus	Plantarflexor group	
**Control**											
3 months old											
Non-trained	31.8 ± 2.5	18.6 ± 0.4	132.4 ± 17.8	18.6 ± 2.0	9.5 ± 1.0	160.5 ± 19.9	7.10 ± 0.84	1.00 ± 0.09	0.51 ± 0.05	8.61 ± 0.93	1.24 ± 0.16
2 days/wk trained	32.1 ± 3.4	18.6 ± 0.2	135.5 ± 13.8	18.3 ± 2.9	8.8 ± 2.7	162.6 ± 15.7	7.29 ± 0.67	0.99 ± 0.16	0.47 ± 0.14	8.75 ± 0.76	1.48 ± 0.05
12 months old											
Non-trained	42.2 ± 6.3†	19.0 ± 0.4†	129.4 ± 7.6	17.3 ± 2.3	9.2 ± 0.9	155.9 ± 9.8	6.80 ± 0.40	0.91 ± 0.11	0.48 ± 0.04	8.19 ± 0.49	1.39 ± 0.19
2 days/wk trained	43.1 ± 4.4†	19.0 ± 0.3†	123.7 ± 12.5	16.0 ± 2.0†	8.7 ± 1.0	148.4 ± 14.5	6.50 ± 0.19†	0.84 ± 0.03†	0.45 ± 0.01	7.80 ± 0.71†	1.34 ± 0.16

Values are expressed as means ± SD. Sample sizes were *N* = 5 to I 0 per group. *Different from comparable non-trained value. †Different from comparable 3-month-old value, *P* < 0.05.

Supplementary Table 6. Muscle fiber cross-sectional area categorized by fiber type for control mice following 2 days per week training.

	Muscle fiber cross-sectional area μm^2				
	I	IIa	IIx	IIb	Total
**Control**					
3 months old					
Non-trained	n/a	961 ± 330	1266 ± 342	2660 ± 297	2122 ± 117
2 days/wk trained	n/a	988 ± 238	1647 ± 247	3063 ± 436	2540 ± 219
12 months old					
Non-trained	n/a	1166 ± 279	1647 ± 506	2639 ± 265	2180 ± 182
2 days/wk trained	n/a	1134 ± 326	1454 ± 141	2557 ± 667	1931 ± 565†

Values are expressed as means ± SD. n/a, data not available because of insufficient number of type I muscle fibers observed in samples to determine values. Sample sizes were *N* = 5 to 6 per group. †Different from comparable 3-month-old value, *P* < 0.05.

Supplementary Table 7. Number of muscle fibers per mm^2 categorized by fiber type for control mice following 2 days per week training. Values are expressed as means ± SD.

	Number of muscle fibers per mm^2				
	I	IIa	IIx	IIb	Total
**Control**					
3 months old					
Non-trained	0.0 ± 0.0	79.3 ± 35.5	82.7 ± 36.7	295.1 ± 69.8	457.2 ± 27.2
2 days/wk trained	0.0 ± 0.0	58.5 ± 21.0	47.4 ± 24.2	272.9 ± 56.7	378.8 ± 27.1
12 months old					
Non-trained	1.0 ± 1.6	87.2 ± 52.5	67.1 ± 33.3	276.4 ± 80.0	431.6 ± 28.3
2 days/wk trained	1.6 ± 3.8	180.0 ± 139.1	87.9 ± 43.7	244.9 ± 43.4	514.3 ± 136.5†

Sample sizes were *N* = 5 to 6 per group. †Different from comparable 3-month-old value, *P* < 0.05.

Supplementary Table 8. Percentage of muscle fibers with dark brown staining (COX^{++} fibers) for control and Snell dwarf mice following 2 days per week training.

	%Cox^{++} fibers
**Control**	
3 months old	
Non-trained	29.9 ± 10.2
3 days/wk trained	33.1 ± 4.6
12 months old	
Non-trained	39.0 ± 11.0
3 days/wk trained	42.1 ± 14.2

Values are expressed as means ± SD. Sample sizes were *N* = 5 to 6 per group. No significant differences were observed.

Supplementary Table 9. Measures of total $CD31^+$ nodes and VCAM-1^+CD31^+ nodes for control mice following 2 days per week training.

	Total $CD31^+/mm^2$	VCAM-1^+CD31^+ nodes/mm^2
**Control**		
3 months old		
Non-trained	632 ± 205	138 ± 95
3 days/wk trained	627 ± 260	*55* ± 49
12 months old		
Non- trained	688 ± 166	92 ± 87
3 days/wk trained	840 ± 68	96 ± 68

Values are expressed as means ± SD. Sample sizes were *N* = 5 to 6 per group. No significant differences observed, $P < 0.05$.
